# Voriconazole Induced Hallucinations and Visual Disturbances in a Female Child: A Case Report and Literature Review

**DOI:** 10.3389/fped.2021.655327

**Published:** 2021-04-23

**Authors:** Rujiang Zheng, Yu Li, Chuyi Guo, Yuxin Pei, Zhiyong Ke, Libin Huang

**Affiliations:** Department of Pediatrics, The First Affiliated Hospital, Sun Yat-sen University, Guangzhou, China

**Keywords:** hallucinations, visual disturbance, voriconazole, children, side-effects

## Abstract

Voriconazole is a second-generation azole widely used for the prevention and treatment of fungal infection in leukemia patients. We report a case of 9-year-old girl with T-cell acute lymphoblastic leukemia who developed hallucinations and visual disturbance after using voriconazole twice. These symptoms began acutely after treatment with voriconazole and resolved rapidly when the voriconazole was stopped. No specific cause was identified, and thus the symptoms were considered to be the adverse drug reactions (ADRs) of voriconazole. Simultaneous development of hallucinations and visual disturbance caused by voriconazole in children rarely have been reported before and the causes of these ADRs are unknown. Several other cases of hallucinations and (or) visual disturbance caused by voriconazole among 15–81 years old patients have been reported in the literature, and are reviewed. Those patients reminded us of the importance of being aware of hallucinations and visual disturbance associated with voriconazole treatment. In addition, we speculate that the hallucinations and visual disturbance are not related to the dosage form of voriconazole. We emphasize that it is also important to monitor the concentration of voriconazole regularly to avoid potential toxicity.

## Background

Invasive fungal infections are major causes of mortality in patients with severer immune-disorder (1, 2). Voriconazole, a second-generation azole of new antifungal agents, may improve the prognosis of patients with invasive fungal infection (3). However, voriconazole is associated with a variety of ADRs, including visual and neurological adverse events (4). Data showed that there are significant differences in the pharmacokinetics of voriconazole in children, adolescents and adults. The trough concentration of voriconazole in children is generally lower, which might be due to the rapid hepatic blood flow, fast metabolism, and first-pass elimination of the drug. Furthermore, the incidence of visual and neurological adverse events in children is lower than that of adults (5). Here, we report a female child who was treated with voriconazole twice to prevent fungal infection after leukemia chemotherapy. These treatments triggered hallucinations and visual disturbance in the patient.

## Patient Presentation

**Figure 1:**
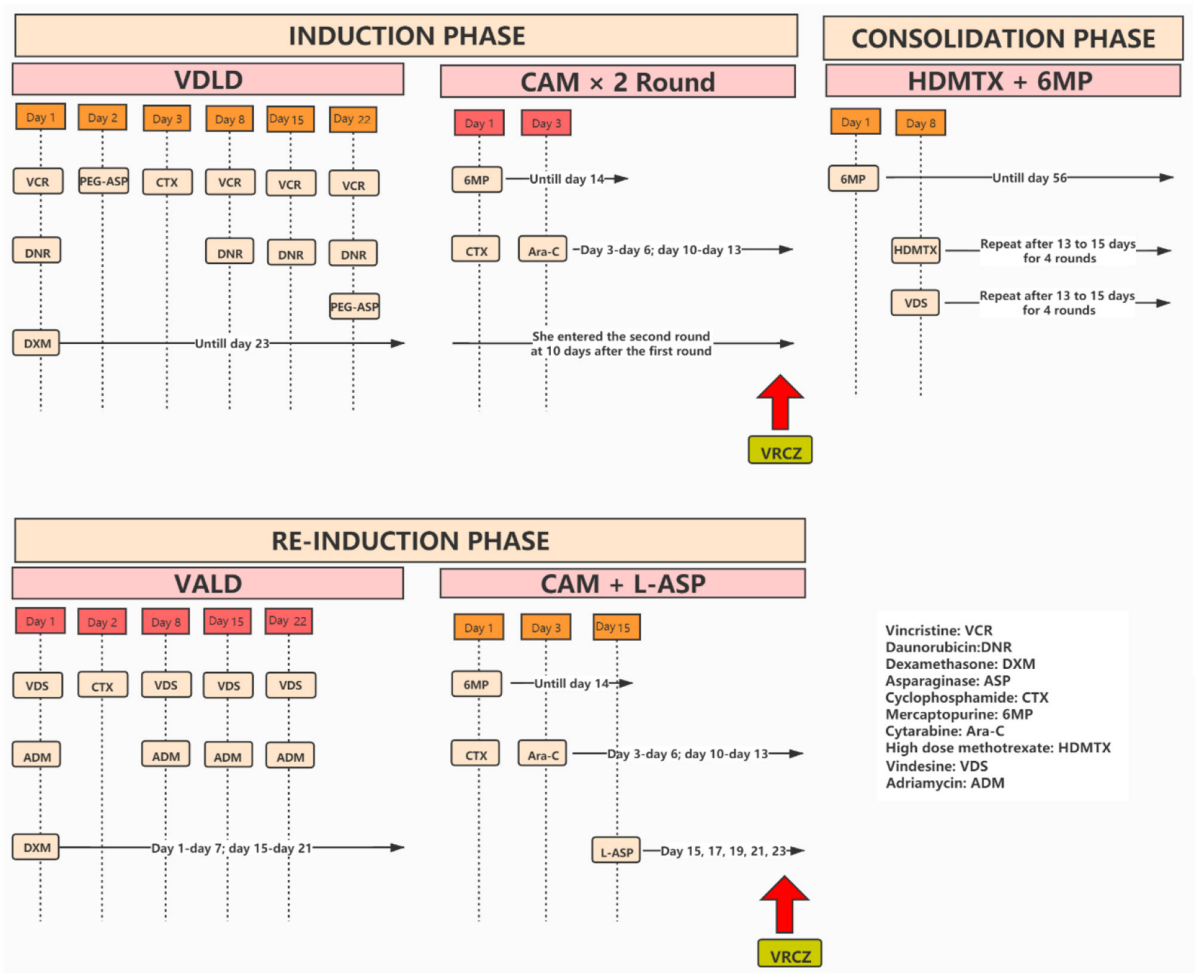
The chemotherapy process in this patient.

A 9-year-old girl was admitted to our hospital with a history of recurrent fever and cough. Physical examination revealed several swollen lymph nodes at her left neck. Blood analysis found higher white blood cells (WBC = 20,800/mm^3^) and anemia (hemoglobin = 9.7 g/dL). Examination of chest CT showed enlarged mediastinal lymph nodes and bilateral pleural effusions. She was prescribed antibiotics, but the fever persisted. A further bone marrow aspiration, morphological examination, immunology, cytogenetics and molecular biology (MICM) tests lead to the diagnosis of intermediate-risk T-cell (III) leukemia. She was hospitalized and treated with South China Children Cancer Group acute lymphoblastic leukemia 2016 (SCCCG-ALL-2016) protocol (Figure 1). In the induction phase of this protocol, dexamethasone, vincristine, daunorubicin, cyclophosphamide, mercaptopurine, cytarabine, L-asparaginase and intrathecal chemotherapy were given. During the induction phase of chemotherapy, she suffered from mental disorder associated with physical disease but these symptoms resolved after treatment with alprazolam and sulpiride. At the end of the first stage of induction phase of chemotherapy, she had episodes of sepsis caused by *Pseudomonas aeruginosa* which were treated with imipenem and teicoplanin. Due to severe neutropenia (<100/mm^3^) and application of broad-spectrum antibiotics, oral voriconazole (200 mg BID, her body weight was 24.4 kg) was administered to prevent fungal infections. On day 3 of voriconazole treatment, she developed hallucinations and visual disturbance. She said that she often scratched the cotton on the top of her head and saw ghosts. The appearance of these symptoms was sudden. The orientation and consciousness of the patient were normal. Although she had mental disorders associated with physical disease, the symptoms did not recur after treatment with alprazolam and sulpiride. The patient had ended a stage of chemotherapy already when symptoms appeared. She had no neurovegetative symptoms and her parents denied their child had previous psychiatric history. Given that the hallucinations and visual disturbance began acutely after the initiation of voriconazole, we thought the symptoms might be caused by voriconazole. When we changed antifungal therapy to caspofungin, the hallucinations and visual disturbance disappeared completely. About 4 months passed, she received re-induction phase which consisted of dexamethasone, vincristine, adriamycin, cyclophosphamide, mercaptopurine, cytarabine, L-asparaginase and intrathecal chemotherapy. Of note, she was given posaconazole to prevent fungal infections when she had severe neutropenia (<100/mm^3^). Because she had pancreatitis, she was fasted and oral posaconazole was discontinued, and voriconazole (200 mg q12h, her body weight was 28 kg) was intravenously administered to prevent fungal infections. On day 2 of voriconazole treatment, she saw demons flying on the ceiling and had visual ghosting again. To this end, we speculated that these symptoms were caused by voriconazole, and the symptoms disappeared immediately after the drug was stopped. The trough concentration of voriconazole on day 3 was 3.6 mg/L.

## Literature Review and Discussion

A review of the literature is presented, with a summary of 7 articles of voriconazole associated hallucinations and (or) visual disturbances in immunocompromised individuals (Table 1). We used the search terms “voriconazole” and “hallucinations” and (or) “visual disturbances” on PubMed to get 28 articles. The title and abstract were reviewed, and 21 unrelated articles were excluded. After carefully reading and reviewing their references, 7 articles were finally confirmed (6–12). Among 7 articles reported, there were 28 males and 15 females; 2 children (all females), 41 adults; There were 40 patients had malignancies, 1 patient had primary immunodeficiency disease (PIDs), 2 patients had aplastic anemia (AA), 1 patient had graft-vs.-host disease (GVHD), 1 patient had stem cell transplantation (SCT), 1 patient had pneumonia and 1 patient had fungemia. The dose of voriconazole was 3–6 mg/kg (Q12H) in only 1 article (6); 200–300 mg/dose (BID) in 4 articles (7–10); 200–400 mg/dose in 2 articles (11, 12). Two articles were used oral formulation (7, 11) and the others were used intravenous formulation of voriconazole (6, 8–10, 12). The patients in 5 articles had visual disturbances (6, 8–12) and in 4 articles had hallucinations (7, 8, 10, 12). Among them, most patients' symptoms appear within a week of taking the voriconazole and resolved after voriconazole withdrawment. However, there were 2 articles described that the symptoms disappear with continued use of voriconazole (6, 12). There were 5 articles that monitored the concentration of voriconazole, which were basically in the normal range (6, 8–11). Our patient's score on the Naranjo Scale suggested that the hallucinations and visual disturbance probably be the ARDs caused by voriconazole (Table 2).

**Table 1:** Literature review of voriconazole induced hallucinations and (or) visual disturbances.

**References**	**Total number of patients**	**diagnosis, y**	**Sex (M: F)**	**VRCZ initial dose/form**	**Drugs interacting with VRCZ**	**Details of symptoms**	**Onset time/duration of symptoms after VRCZ stop, days**	**Plasma VRCZ trough level (μg/mL)**
Zonios et al. (6)	12	Breast cancer/MDS, myelofibrosis/CMML/ALL/AML/NHL, 33–68	9:3	3–6 mg/kg Q12H, iv	NA	Visual and/or auditory	1–8/1–5 or treatment continued	5.26 ± 0.83
Agrawal and Sherman (7)	1	Acute myelogenous leukemia, 78	Male	300 mg bid, po	Idarubicin, cytarabine, verapamil	Hearing Christmas music	2/3	NA
Imhof et al. (8)	6	AML/SCT/AA, 43–78	5:1	200–300 mg BID, iv	Cyclosporine, mmf, pred	Hypotonia, anxiety, insomnia, visual hallucinations, irritability,	NA/3–4	1.5–6.4
Gaies et al. (9)	1	AA, 38	male	300 mg BID, iv	NA	Auditory hallucination, temporo-spatial disorientation.	7/2	7.5
Hideo et al. (10)	6	ALL/PIDs/pneumonia/fungemia/cervical cancer, 15–81	3:3	200–300 mg BID, iv	NA	Visual disturbance, hallucinations, shame	2–42/0–2	4.25 ± 2.25
Sakurada et al. (11)	15	hematological malignancies, 52–78	9:6	200–400 mg BID, iv/po	Omeprazole, rabeprazole, prednisolone, dexamethasone	Blurred vision, color blindness, photophobia, diplopia	1–7/2–12	5.40 ± 2.37
Bayhan et al. (12)	1	ALL, 16	Female	400 mg BID, iv	Prednisolone, vincristine, daunorubicin, L-asparaginase, methotrexate	Visual and auditory hallucinations and disturbance.	4/resolved completely 4 days later without VRCZ withdrawment	NA
Our patient	1	ALL, 9	Female	200 mg BID, iv/po	Dexamethasone, vincristine, daunorubicin, dexamethasone, asparaginase, adriamycin	Hallucinations and visual disturbances	2–3/1	3.6

*VRCZ, voriconazole; CMML, chronic myelomonocytic leukemia; GVHD, graft-vs.-host disease; IV, intravenous; MDS, myelodysplastic syndrome; NHL, non-Hodgkin lymphoma; SCT, stem cell transplantation; PO, per os; ALL, acute lymphoblastic leukemia; AA, aplastic anemia; PIDs, primary immunodeficiency disease; MMF, mycophenolate mofetil; pred, prednisone*.

**Table 2:** The weighted scores of our patient on the Naranjo Scale (13).

**To assess the adverse drug reaction, please answer the following questionnaire and give the pertinent score**	**Yes**	**No**	**Do not know**
1. Are there previous conclusive reports on this reaction?	+2		
2. Did the adverse event appear after the suspected drug was administered?	+2		
3. Did the adverse reaction improve when the drug was discontinued or a specific antagonist was administered?		0	
4. Did the adverse reaction reappear when the drug was readministered?	+2		
5. Are there alternative causes (other than the drug) that could on their own have caused the reaction?	+1		
6. Did the reaction reappear when a placebo was given?			0
7. Was the drug detected in the blood (or other fluids) in concentrations known to?		0	
8. Was the reaction more severe when the dose was increased, or less severed when the dose was decreased?			0
9. Did the patient have a similar reaction to the same or similar drugs in any previous exposure?	+1		
10. Was the adverse event confirmed by any objective evidence?			0
Total score	8		

*The total score ≥ 9 was empirically defined as “definitely” having caused the ADR; the total score 5–8 was defined a “probably” caused the ADR; the total score 1–4 was defined a “possibly” and total score ≤ 0 indicated association with drug was “doubtful”*.

Voriconazole is a broad-spectrum antifungal drug that has been shown to be effective in treating fungal infections such as aspergillus (14). Voriconazole is metabolized by the CYP450 system in the liver, mainly by CYP2C9, CYP3A4, and CYP2C19 (15). It can reach higher concentrations in the cerebrospinal fluid by traversing the blood-brain barrier (16, 17). Voriconazole was found to be associated with some ADRs (18). An analysis of a French pharmacovigilance database (19) showed that ADRs of voriconazole included liver function test abnormalities (23%), visual disturbances (18%), rashes (17%), neurologic disturbances (14%), cardiovascular events (10%), hematologic disorders (8%), and renal disturbances (4%). Other less commonly identified ADRs included headache, nausea, vomiting, and diarrhea. Simultaneous development of hallucinations and visual disturbance caused by voriconazole in children rarely have been reported before. The mechanism through which voriconazole-induced hallucinations and visual disturbance is not clear.

Voriconazole is available as oral and intravenous formulations. For the patients of 2–12 years of age, the recommended dosing regimen for voriconazole in the European is 7 mg/kg BID (for the intravenous formulation) and 200 mg BID (for the oral formulation), without loading doses. That recommended dosing regimen of voriconazole can result in doses in excess of 15 mg/kg in the youngest children. This may be due to the significantly reduced bioavailability and the increased clearance rate of voriconazole in children relative to adults (20). Compared to adults, data show that, the children <12 years have approximately a 50% reduction in bioavailability for voriconazole, which suggests that children need higher doses of voriconazole to against fungal infection (21, 22). In the present case, the patient developed hallucinations and visual disturbance suddenly after using voriconazole twice (7–8 mg/kg BID) and these symptoms disappeared immediately when the drug was stopped. It has been reported that the incidence of hallucinations is affected by formulation of voriconazole. For instance, hallucinations are associated with intravenous formulation and are resolved when voriconazole is given as oral formulation (6). However, in our patient, hallucinations and visual disturbance occurred regardless of oral or intravenous formulation. Therefore, we speculated that the occurrence of hallucinations and visual disturbance of voriconazole might be related to the concentration of the drug in plasma.

It is reported that voriconazole trough concentration > 1 ug/mL is related to good clinical response to treatment and improved survival rate. Voriconazole binds to plasma proteins and the concentration in cerebrospinal fluid is about 30–60% of its concentration in plasma. High level of voriconazole in cerebrospinal fluid may cause changes in the retina and central nervous system, leading to hallucinations and neurological diseases (7, 23). Data has suggested that high concentration of voriconazole in plasma may increase the risk of hallucinations and neurologic adverse effects (8, 9). Kato et al. (10) observed 123 patients treated with voriconazole and found that the trough concentration of voriconazole in patients who suffered from visual disturbance was higher while patients with central symptoms had similar trough concentration of voriconazole with those who did not develop adverse reactions to voriconazole. A systematic review demonstrated that when the trough concentrations of voriconazole were more than 4.0 mg/L, the risk of neurotoxicity increased (24). However, Chu et al. (25) retrospectively reviewed 108 patients who received voriconazole and found that the trough concentration of voriconazole of >5.5 mg/L was not associated with increased incidence of encephalopathy. Sakurada et al. (11) also found no significant difference between the concentrations of voriconazole in plasma of patients who had or did not have visual hallucinations. In our case, there was no significant drug interactions that could alter the concentration of voriconazole in plasma. The trough concentration of voriconazole was not obtained for our patient when the hallucinations and visual disturbance occurred for the first time, and the voriconazole concentration was 3.6 mg/L when the hallucinations and visual disturbance occurred for the second time. Further studies are needed to further confirm whether the occurrence of hallucinations and visual disturbance is dependent on the concentration of voriconazole. Elsewhere, Imhof et al. (8) found that adverse neurological symptoms resolved spontaneously during voriconazole treatment. Bayhan et al. (12) reported a patient who developed hallucinations after 4 days of intravenous voriconazole treatment and the symptoms completely disappeared after 4 days without adjusting the dosage and formulation of voriconazole. We propose that the relationship between the withdrawal of voriconazole and the disappearance of hallucinations deserves further observation.

In a conclusion, the present case report has revealed that hallucinations and visual disturbance associated with voriconazole treatment are not rare and such cases should be monitored in clinical practice. We should be aware of hallucinations and visual disturbance associated with voriconazole treatment and initiate prompt treatment for these conditions. In addition, we speculate that the hallucinations and visual disturbance might not be related to the dosage form of voriconazole. Patients should be informed of the possibility of these adverse events before starting voriconazole treatment. Although the relationship between the occurrence of hallucinations and visual disturbance and concentration of voriconazole is unclear, it is important to monitor the concentration of voriconazole regularly to avoid potential toxicity.

## Data Availability Statement

The original contributions presented in the study are included in the article/supplementary material, further inquiries can be directed to the corresponding author/s.

## Ethics Statement

Written informed consent was obtained from the individual(s), and minor(s)' legal guardian/next of kin, for the publication of any potentially identifiable images or data included in this article.

## Author Contributions

LBH and RJZ conceptualized and designed the study, reviewed, and revised the manuscript. YL carried out the initial analyses and drafted the initial manuscript. ZYK and LBH coordinated and supervised the data collection. YL, CYG, and YXP critically reviewed the manuscript. All authors read and approved the final manuscript.

## Conflict of Interest

The authors declare that the research was conducted in the absence of any commercial or financial relationships that could be construed as a potential conflict of interest.
